# Genetic Variability of *IFNAR2* Gene and Risk of COVID-19 Critical Illness Among the Tunisian Population

**DOI:** 10.3390/v18101149

**Published:** 2026-10-16

**Authors:** Abir Azri, Duaa A. Althumairy, Hager Memni, Mohammed A. Almalki, Ikbel Hadj Hassine, Noureddine Chatti, Jawhar Gharbi, Manel Ben M’hadheb

**Affiliations:** 1 LR26ES05: Viral Genomics, Architecture and Functions of Macromolecules, Higher Institute of Biotechnology, University of Monastir, B.P. 74, Tahar Haddad Street, Monastir 5000, Tunisia; abirazri2020@gmail.com (A.A.); noureddinealichatti@gmail.com (N.C.); 2 USCR-SAG: Genetic Sequencing and Analysis, Higher Institute of Biotechnology, University of Monastir, B.P. 74, Tahar Haddad Street, Monastir 5000, Tunisia; 3 Department of Biological Sciences, College of Science, King Faisal University, P.O. Box 380, Al-Ahsa 31982, Saudi Arabia; dalthumairy@kfu.edu.sa (D.A.A.); malmalki@kfu.edu.sa (M.A.A.); 4 Salem Ben Hassen Medical Analysis Laboratory, Avenue of 18 January 1952, Monastir 5000, Tunisia; hagermemni@yahoo.fr; 5 Department of Virology, Health Sciences College, Western University, 1393 Western Road, London, ON N6G 3K7, Canada; ihadjhas@uwo.ca

**Keywords:** IFNAR2, human genetic factors, polymorphisms, critical COVID-19

## Abstract

The *IFNAR2* gene encodes an essential component of the type I interferon receptor, which is essential for antiviral defense. It has been proven that the severity of COVID-19 may be influenced by the genetic variants in *IFNAR2*; however, the results are inconclusive in the African cohorts. Thus, in this study, we investigate the association between three *IFNAR2* SNPs and the critical form of COVID-19 in the Tunisian population. A total of 413 COVID-19 patients classified as hospitalized with critical illness (ICU) (*n* = 46) and non-hospitalized (*n* = 367) are included. Genotyping of three *IFNAR2* polymorphisms (rs1051393, rs2236757, rs9984273) was performed using Taq Man RT-PCR assays. rs2236757 (OR = 0.31, 95% CI: 0.16–0.60), *p* = 4 × 10^−4^) and rs9984273 (OR = 0.41, 95% CI: 0.22–0.76, *p* = 0.0055) were associated with COVID-19 severity with a potential protective effect. However, rs1051393 did not show a significant association with disease severity. Additionally, exploratory haplotype analysis suggested a possible protective association for the TAC haplotype (OR = 0.11, 95% CI: 0.02–0.86, *p* = 0.036), based on a very small number of carriers and not significant after correction for multiple testing. The GAC haplotype, absent from ICU patients, yielded a continuity-corrected OR of 0.03 (95% CI [0.002–0.53]); this estimate rests on a zero cell and is hypothesis-generating only. These preliminary findings, obtained from a single-center cohort with a limited number of critically ill patients, warrant replication in larger, independent, and multicentric populations.

## 1. Introduction

Severe acute respiratory syndrome coronavirus 2 (SARS-CoV-2) is a new coronavirus that emerged in December 2019 in Wuhan, China, and is responsible for the global outbreak of coronavirus disease 2019 (COVID-19). SARS-CoV-2 is an enveloped virus, a positive-sense single-stranded RNA virus belonging to the *Coronaviridae* family [1]. Up to 12 July 2026, over 779 million confirmed cases and over seven million deaths have been reported worldwide [2]. According to the Tunisian Ministry of Health, in Tunisia, the first case of COVID-19 was reported on 2 March 2020, with a rapid increase in cases of infection, peaking in 2021 and 2022 [3]. In Tunisia, 1,153,023 confirmed COVID-19 cases, including 29,423 deaths, were reported by the WHO as of 12 July 2026 [4].

COVID-19 is extremely contagious; it is transmitted basically through respiratory droplets or direct contact, while fecal-oral transmission is also possible [5]. The virus’s incubation period takes about 2 to 14 days, and the symptoms appear 4 to 7 days later [6]. It has been shown that the symptoms can vary from one individual to another from mild to severe and critical infection, suggesting that both environmental and genetic risk factors may influence the COVID-19 progression [7]. The severity of COVID-19 depends on several factors, such as advanced age (over 65), ethnicity, gender (males), and comorbidities (e.g., diabetes, obesity, hypertension, and cardiovascular diseases) [8,9]. However, severe symptoms have been reported in young patients who do not suffer from any of these risk factors and without any significant medical history [10]. This suggests that the immune response to SARS-CoV-2 infection may be influenced by intrinsic genetic factors [11]. In this context, several research studies have reported the involvement of genetic polymorphisms (SNPs) in COVID-19 susceptibility and severity [12,13]. Among the polymorphisms that are deeply studied for their implication in COVID-19 severity, researchers have identified SNPs in the *IFNAR2* gene [14,15]. The latter plays a key role in the antiviral immune response [16]. These single nucleotide variations may change the receptor’s structure, the IFN binding site, and the gene expression, thereby potentially affecting disease severity [17]. A genetic association study conducted in a Palestinian cohort demonstrated a strong association between the rs2236757 variant of *IFNAR2* and severe forms of COVID-19, where the A allele was associated with worsening disease, particularly with an increased incidence of dyspnea (OR = 4.70) and intensive care hospitalization [18]. Another research study conducted by Fricke-Galindo and his collaborators on 1202 patients showed that variants rs2236757, rs3153, rs1051393, and rs2834158 are associated with an increased risk of mortality in the Mexican population [15]. On a large scale (GenOMICC study), a GWAS conducted on 2244 critically ill patients in the United Kingdom revealed a strong association (*p* = 4.99 × 10^−8^) between rs2236757 and critical forms of the disease [13]. However, these results have not been widely explored in North African populations, in particular, Tunisia, which has a specific genetic diversity that could impact and modulate the immune response to SARS-CoV-2. So, the aim of this study is to identify the genetic variants in this gene that are associated with worse COVID-19 outcomes in a differentiated genetic context.

## 2. Materials and Methods

### 2.1. Ethics Statement

The study was carried out in accordance with Tunisian law concerning the protection of personal data (Organic Law No. 2004-63 of 27 July 2004, relative to the Protection of Personal Data) and the ethical guidelines for biomedical research in Tunisia. According to the Declaration of Helsinki (paragraph 32) [19] and the International Ethical Guidelines for Health-Related Research of CIOMS [20], it is possible to carry out retrospectively a study based on coded, anonymized, and residual biological material collected during routine diagnostic care, when the study has been approved by a competent ethics committee, as was the case for this study. This is a retrospective genetic association study realized using biological leftovers from whole blood samples collected during the period from March to August 2021 during routine diagnostic care at the Medical Analysis Laboratory Ben Hassan Salem. No additional blood was collected from the participants for this study. The participants had given their written informed consent at the moment of collection of the initial sample allowing the use of their clinical data and their residual samples for research purposes. Biological samples and clinical data were then coded and anonymized before being analyzed.

In addition, appropriate steps were taken to ensure the confidentiality of the participants. The formal ethics approval for the realization of this retrospective genetic association study, i.e., the secondary analysis of these residual, coded, and anonymized samples, was obtained in September 2023 from the institutional ethics committee (CERSVS/ISBM 008/2023).

### 2.2. Study Design and Cohorts

This research study included 413 patients with a confirmed SARS-CoV-2 infection RT-PCR test or a positive nasopharyngeal test between March 2021 and August 2021. All the participants were unvaccinated against COVID-19 and aged over 18 years. No co-infection was documented in the available clinical and laboratory records; however, systematic microbiological screening for viral, bacterial, or fungal co-infection was not performed, and undocumented co-infections cannot be excluded. These blood samples were obtained from the Ben Hassan Salem Medical Analysis Laboratory.

Patients were classified into two groups: non hospitalized group with mild symptoms and hospitalized group with critical illness (hypoxemic pneumonia requiring oxygen therapy and mandatory admission to an intensive care unit (ICU), *n* = 46). At the time of enrollment, clinical and demographic data, such as age, sex, and comorbidities, were collected via a well-structured questionnaire supervised by healthcare personnel.

Ethical approval and consent procedures for this study are detailed in Section 2.1 (Ethics Statement).

### 2.3. DNA Extraction and Quantification

Using the conventional method of salting-out [21], the DNA was extracted from the whole blood samples on EDTA with the following steps. The first step consists of red blood cell lysis using a hemolytic solution (140 mM NaCl, 8.25 mM Tris, pH 7.2, Biolab Diagnostics, Italy). After centrifugation, the retained leukocyte pellet was resuspended in a lysis solution (20 µL Tris (1 M, pH 7.2), 20 µL NaCl (5 M), 40 µL EDTA (0.5 M, pH 8), 20 µL proteinase K (20 mg/mL), 120 µL SDS 10%, and ultrapure water, Biolab Diagnostics, Italy) and incubated overnight at 37 °C. Then, to precipitate the proteins, the obtained solution was salted out at 4 °C using a saturated NaCl solution. Finally, The DNA in the supernatant fluid was precipitated using concentrated solution of ethanol, then dissolved in ultrapure water and stored at −20 °C until further use.

By measuring absorbance at 260/280 nm, the DNA concentration and purity were determined spectrophotometrically using a NanoDrop 2000 (Thermo Fisher Scientific, Waltham, MA, USA). DNA integrity was verified by agarose gel electrophoresis.

In order to ensure that each genotyping run was free of false positive results, no-template (water) controls were run with each genotyping run. Approximately ten percent of all samples were genotyped in duplicate and showed 100% agreement. Although we did not include any external reference genotyping standards (i.e., HapMap/1000 Genomes control DNA) in our study, this should be taken into account in future studies.

### 2.4. TaqMan Based Genotyping

The rs1051393, rs2236757, and rs9984273 SNPs at the *IFNAR2* gene were genotyped using a real-time PCR approach with a TaqMan probe from Applied Biosystems (Carlsbad, CA, USA). The primers, probes, and Master Mix (40× were given by Thermo Fisher Scientific (Waltham, MA, USA). The probes’ sequences were designed as [VIC/FAM]: GCTTTTGAGCCAGAATGCCTTCATC**[G/T]**TCAGATCACTTAATTTGGTTCTCAT for rs1051393, CAAATCCCAAAAGAGATTAAGGCCT**[A/G]**CCTCTAAATGAAATTCTCAGTCTTA for rs2236757, and TAAAGAGCAAACAGTACAGCTGATA**[C/T]**GGACTCTCTCTCTCTTTTTTTTTTT for rs9984273.

Each reaction contained 0.5 μL of primer/probe mixture, 5 μL of Master Mix, 3.5 μL of nuclease-free water, and 1 μL of purified DNA. The PCR program was as follows: 10 min at 95 °C for the first denaturation, then 40 cycles (15 s at 95 °C for the second denaturation and 1 min at 60 °C for primer annealing). *IFNAR2* SNPs were selected based on previous studies that have reported an association between these polymorphisms and COVID-19 severity. The overall genotyping call rate across all three variants exceeded 98.5%, with no individual sample missing more than one genotype.

A post hoc power analysis suggests that the study had little statistical power to identify associations between the modest effect sizes observed in this study (*n* = 46 in the ICU group) and those identified here. The study was underpowered for rare genotypes categories and haplotypes. This was considered when interpreting all of the identified associations, which are considered exploratory rather than confirmatory.

### 2.5. Statistical Analysis

All statistical analyses were performed with the use of SPSS version 23 and the SNPStats online platform [22]. In each group, the Hardy–Weinberg equilibrium of allele frequencies of the three variants were evaluated via a chi-square test with one degree of freedom (*p* > 0.05 in both groups). The relationship between COVID-19 severity and the *IFNAR2* allele frequency was assessed with a Fisher’s exact test on 2 × 2 contingency tables. The crude odds ratios (OR) and 95% confidence intervals from these analyses are presented in Table 1. Logistic regressions were conducted under three genetic models (codominant, dominant, recessive) and their associated age/sex-adjusted odds ratios for each SNP using the SNPStats platform (Catalan Institute of Oncology ICO, Catalonia, Spain). Selection of the model that best fit the data was determined by the lowest Akaike information criteria (AIC) and Bayesian information criteria (BIC) among these three models, a common practice within the SNPStats pipeline that is applied equally to all three SNPs before evaluating associations. This method is preferred to post hoc, result-dependent decision making. Although the codominant model was included in Table 2 for transparency purposes, it was not the chosen model for any of the three SNPs. The obtained *p*-values were adjusted for multiple comparisons through the Benjamini–Hochberg procedure [23] to manage the false discovery rate (FDR). Haplotype odds ratios were computed from haplotype chromosome counts relative to the reference haplotype TGT, and the *p*-values of the seven haplotype comparisons were adjusted using the Benjamini–Hochberg procedure.

For each polymorphism, the ancestral allele was determined using the Ensembl database [24], and the risk alleles were defined based on statistical association with COVID-19 severity. To explore the linkage disequilibrium (LD) structure and the combined effect of several genetic variants, an haplotypic analysis was performed using the SNPStats online software (https://www.snpstats.net/start.htm, 5 June 2026). It estimates haplotype frequencies based on the expectation–maximization (EM) algorithm. D′ and r^2^ statistics were used to evaluate linkage disequilibrium. For rare haplotypes with zero observed counts in contingency cells, such as GAC, the Haldane–Anscombe continuity correction [25,26] (adding 0.5 to all cell counts) was applied to ensure finite and stable odds ratios and 95% confidence intervals.

## 3. Results

### 3.1. Clinical and Demographic Characteristics of COVID-19 Participants

This research study included 413 participants stratified into 367 non-hospitalized cases and 46 hospitalized cases. Starting with age as a first characteristic, the mean age in the non-hospitalized cohort was slightly higher (51.5 ± 16.65) compared to the hospitalized cohort (48.5 ± 17.2). However, this difference did not reach statistical significance according to Student’s *t*-test (*p* = 0.251). Fisher’s exact test did not reveal a statistically significant difference (*p* = 0.871) in sex distribution between the two groups. However, females were more represented in both groups: (65.9%) in the non-hospitalized group and (67.4%) in the hospitalized. Additionally, the lifestyle factors (tobacco, hookah, and alcohol) do not reveal a statistically significant association with the critical form of COVID-19. Regarding comorbidities, all of them showed no significant difference between the two cohorts (Table 1).

### 3.2. Association of IFNAR2 Variants with COVID-19 Severity

We evaluated the genetic association between three polymorphisms (rs1051393, rs2236757, and rs9984273) located in the *IFNAR2* gene and the critical form of COVID-19 in the cohort of 413 patients. For both cohorts (non-hospitalized and hospitalized), the genotype distribution of the three variants was in Hardy–Weinberg equilibrium (*p* > 0.05): for the non-hospitalized cohort (rs1051393, *p* = 0.084; rs2236757, *p* = 0.19; rs9984273, *p* = 0.35) and for the hospitalized cohort (rs1051393, *p* = 0.72; rs2236757, *p* = 0.41; rs9984273, *p* = 0.73). The genetic association between the three SNPs and the critical COVID-19 form was evaluated first with Fisher’s exact test. The results shown a statistically significant difference in genotype frequencies between non-hospitalized and hospitalized groups for rs2236757 (OR = 0.35, 95%CI: 0.19–0.67, *p* = 0.001) and rs9984273 (OR = 0.39, 95%CI: 0.21–0.73, *p* = 0.0033). Then, a logistic regression analysis under three genetic models adjusted for age and sex was performed to estimate the association between each variant and the critical COVID-19 form. A Benjamini–Hochberg correction method (FDR) was also applicated for the three SNPs and the three models (codominant, dominant, and recessive).

Adjustment was limited to age and sex because, with only 46 events, the number of covariates that can be reliably included in a logistic regression model is restricted by the risk of overfitting (a commonly used rule of thumb states that at least 10 events are required per covariate). Comorbidities and lifestyle factors (tobacco, hookah, alcohol) were assessed univariately (Table 1) and showed no statistically significant association with disease severity in this cohort; they were therefore not included as additional covariates in the adjusted model, and the estimates obtained after adjusting for age and sex should not be interpreted as having been adjusted for all potential confounders.

For rs2236757, a statistically significant difference in genotype frequencies between non-hospitalized and hospitalized groups was reported according to the three tested models. According to the low Akaike information criteria (AIC) and Bayesian information criteria (BIC), the dominant model was selected for rs2236757 to explain the association with COVID-19 severity (AIC = 282.6; BIC = 298.7). Using GG as a reference genotype that is more frequent in hospitalized patients (63% vs. 37.6%), the AA + AG genotype was significantly less frequent in hospitalized cases (37% vs. 62.4%) and was associated with the illness severity with a protective effect against COVID-19 critical forms (OR = 0.31, 95%CI: 0.16–0.60, *p* = 4 × 10^−4^). After Benjamini–Hochberg correction, the association was still statistically significant (FDR = 0.0036). So, the rs2236757A minor allele is associated with a less severe form of COVID-19.

The dominant model was the best one to describe the association of rs9984273 and the disease severity (AIC = 287.6, BIC = 303.7). There is a significant difference in the genotype distribution between the two groups (*p* = 0.0055) where the CC + TC genotypes are less frequent in the hospitalized cohort (50% vs. 71.9%) and statistically associated with a protective effect against worse COVID-19 outcomes (OR = 0.41, 95%CI: 0.22–0.76, *p* = 0.0055), and even after Benjamini–Hochberg correction, the association was still statistically significant (FDR = 0.0165), whereas no significant association was found between the genotypes and alleles of rs1051393 (Table 2).

An interaction analysis was conducted to explore whether sex modifies the association between each variant and disease severity. With an age adjustment, no statistically significant interaction between rs2236757 and the COVID-19 critical form (*p* = 0.36) was observed. This is also likely for rs9984273 (*p* = 0.36) and for rs1051393 (*p* = 0.3).

### 3.3. Linkage Disequilibrium and Haplotype Analysis

Given the possible combined impact of the three tested *IFNAR2* variants (rs1051393, rs2236757, and rs9984273), a linkage disequilibrium (LD) and haplotype analysis were performed. No LD was reported between rs2236757 and rs9984273 (D′ = 0.0741, r^2^ = 0.0028, *p* = 0.12), nor between rs9984273 and rs1051393 (D′ = 0.0524, r^2^ = 0.0012, *p* = 0.31), whereas a low LD was observed between rs2236757 and rs1051393 (D′ = 0.4648, r^2^ = 0.18, *p* < 0.001).

Since there was no evidence of strong linkage disequilibrium (LD) among rs2236757/rs9984273 and rs1051393, and weak LD existed between rs2236757 and rs1051393, the biological interpretation of the haplotype analyses using all three markers is limited. The resulting haplotypes are likely to be a consequence of random segregation of each marker’s allele(s), rather than representing a single functional unit. Thus, the observed effects of the TAC and GAC haplotypes should be considered as a starting point for generating hypotheses and not as conclusive evidence of a functionally relevant haplotypic effect.

In this studied population, the haplotype analysis identified eight distinct haplotypes, with TGT and TGC being the most frequent ones (25.99% and 24.67%, respectively). The overall haplotype distribution differed significantly between non-hospitalized and hospitalized cases (global haplotype association test, *p* = 0.00018), indicating that haplotype frequencies as a whole differ according to disease severity (Table 3).

Using TGT as the reference haplotype, TAC was the only individual haplotype that reached nominal significance, with a lower frequency among hospitalized cases (1.25% vs. 7.40%) and a statistically significant association with a decreased risk of severe disease (OR = 0.11, 95% CI: 0.02–0.86, *p* = 0.036). However, the estimation of this effect is based on a very small number of individuals carrying the TAC haplotype in the hospitalized group, which explains the wide confidence interval; thus, this finding may be considered exploratory rather than confirmative. Furthermore, this specific comparison did not remain significant after adjusting for multiple testing over the eight haplotypes evaluated. Therefore, the results of this specific TAC comparison cannot be considered to have been “confirmed” by the global haplotype test mentioned previously. The reason for this is that each of these tests examines different statistical hypotheses; the global test examines whether there exists a general distribution difference between all eight haplotypes, while the TAC comparison examines whether there is a significant difference between the TAC haplotype and the reference haplotype. The other remaining haplotypes did not show an association with disease severity (Table 3). Of note, the GAC haplotype has never been found among hospitalized patients (0%) and was only present in 10.36% of non-hospitalized cases. With a Haldane–Anscombe continuity correction, we obtained a finite yet imprecise estimate (OR = 0.03, 95% CI: 0.002–0.53, *p* = 0.00001), which reflected the lack of data for this haplotype within the ICU group as opposed to evidence of either a protective or risk effect. Therefore, this result cannot be over-interpreted and needs to be confirmed by a larger cohort.

## 4. Discussion

Type I interferons are essential for an early and efficient antiviral immune response [27]. A genetic variation in *IFNAR2* gene coding for a subunit of the type I interferon receptor may critically influence the COVID-19 outcome by modulating interferon signaling efficiency.

Our study showed a significant association of rs2236757 with COVID-19 severity, with the A allele specifically more frequent in non-hospitalized cases. This finding is in line with Mahnaz Safari and his collaborators’ study that reported a reduction of the disease severity in the presence of the rs2236757A allele, suggesting a protective effect of this latter compared to the G allele [28]. However, these results contradict multiple other studies; one such example is a large-scale Brazilian population study where the AA genotype had a significantly higher risk of ICU admission [11]. The conflicting findings and potential reasons for them will be further elaborated upon below.

A major inconsistency needs to be examined: the protective relationship identified in our study between rs2236757-A and the development of severe disease was in the opposite direction from that reported by GenOMICC study, which used Mendelian Randomization to demonstrate that reduced levels of *IFNAR2* were associated with an increased risk of developing life-threatening illnesses [13]. Likewise, a similar inconsistency was observed in the Palestinian cohort, where rs2236757-A was associated with an increased risk of severe COVID-19 (OR = 4.70 for dyspnea) [18]. Similarly, in the Mexican cohort, a higher rs2236757 minor-allele (A) frequency was observed among non-survivors, indicating that A behaves as a risk allele in that population, consistent with the direction reported by GenOMICC [15]. Conversely, our demonstration of a protective effect for rs2236757-A is consistent with an additional ARDS/COVID-19 cohort who also demonstrated lower mortality rates among carriers of both the rs2236757-A and rs9984273-C minor alleles, even though they acknowledged the contradictory results reported by the GenOMICC study [29].

In addition, it is interesting to note that rs2236757-A has been linked to increased *IFNAR2* expression in at least one study [30] (individuals with the GG genotype had significantly less *IFNAR2* expression than AA/AG carriers) and thus could, if replicated, be compatible with a protective function of the A allele. As such, the varying directions of association for the same variant across different populations and cohorts, referred to as “flip-flop” associations in the genetics literature, may be due to varying degrees of linkage disequilibrium with an unmeasured causal variant across ethnic groups, population-specific interactions between genes and environments, or merely chance findings from underpowered studies. Since our hospitalized cohort was relatively small in number, we cannot rule out that this difference is simply a result of random error and are presenting this inconsistency openly as a source of considerable uncertainty regarding whether this represents a true population-specific protective mechanism. We have also confirmed that our allele coding (A/G) is in accordance with the forward-strand convention reported in dbSNP (build GRCh38) [31], so we can eliminate a simple strand-orientation artifact as an explanation for this inconsistency. Therefore, a larger, sufficiently powered Tunisian or North African cohort will be necessary to replicate this finding prior to it being deemed credible.

In addition to the biological/expression-based and ancestry/linkage-disequilibrium hypotheses outlined above, there are two other, non-exclusive methodological considerations that may also account for the cross-population variability observed in COVID-19 genetic associations. First, phenotype definition varies greatly across studies. For example, GenOMICC investigators and Fricke-Galindo et al. studied mortality/critical illness in large, multicenter, prospectively recruited cohorts. The Palestinian and Tunisian studies were smaller and single-center and employed different criteria to define disease severity (e.g., ICU admission with hypoxemic pneumonia vs. in-hospital death). Second, study design differences (genome-wide association vs. candidate gene approach, prospective vs. retrospective recruitment) impact statistical power and vulnerability to selection bias differently. When combined with the unknown level of population stratification and the unrepresentative nature of the hospitalized subgroup described elsewhere in this paper, these factors additionally hinder direct comparison with larger, more powerful studies.

Although rs2236757 is an intronic variant, it may potentially contribute to modulating gene expression levels by influencing mRNA splicing efficiency, regulatory elements, or transcription factor binding that can possibly result in modifications of receptor abundance and thus type I interferons signaling pathways. In this context, a recent study revealed a significant decrease in *IFNAR2* levels in patients with GG genotype compared to patients with AA and AG genotypes [30]. These mechanisms were not tested in our cohort and are offered only as hypotheses. If confirmed, reduced receptor expression could limit interferon signaling and weaken the early antiviral response, but our data cannot establish such a link.

rs9984273 is a 3′UTR polymorphism of the *IFNAR2* gene; this region can be involved in post-transcriptional regulation [32,33]. Our study reveals an association of the allele C with a protective effect against the critical form of COVID-19. This is in correlation with the findings of Jalkanen and collaborators, who reported a less severe form of COVID-19 in patients with the allele C [29]. Whether this variant has any functional effect on *IFNAR2* expression was not assessed here, and the observed association may instead reflect linkage with another, unmeasured variant. Functional studies would be required to explore any biological role of this variant.

rs1051393 is a missense variant that changes phenylalanine to valine in the signal peptide region of *IFNAR2* [31,34]. We found no association between this variant and disease severity. This may reflect a true absence of effect or, given the small ICU subgroup, insufficient statistical power; we did not assess its functional consequences, and we draw no conclusion about its effect on protein trafficking or receptor function.

Due to the inadequate statistical power of the hospitalized/ICU subgroup (*n* = 46) for identifying an association between tobacco, alcohol, hookah, and comorbidities with severe COVID-19 based upon demographic and clinical data, the current study, a single-center, underpowered dataset, cannot be used to determine if genetic variation is more influential in determining the severity of COVID-19 than clinical or environmental factors in the Tunisian population. Furthermore, there are some demographic characteristics of this cohort that differ from those that have been reported to date as risk factors for COVID-19 severity: patients admitted to the hospital/ICU were somewhat younger than those not hospitalized, female patients comprised a larger percentage of the patients in both groups, and none of the traditional clinical risk factors (hypertension, diabetes, cardiovascular disease, tobacco use) were found to be associated with severity. The fact that many studies report higher rates of male prevalence and comorbidity burden in COVID-19 severity studies throughout the world is in contradiction to the results of this study. The probable explanation for the inconsistencies in these results is the limited sample size and single-center design of the hospitalized subgroup, as well as the referral and admission policies implemented during the study period. However, it should be noted that these results do not necessarily indicate a reversal of previously documented risk factors.

Beyond the findings of the individual SNP associations, the exploratory haplotype analysis (Section 3.3) provides additional hypothesis-generating insight. The TAC haplotype occurred less frequently in hospitalized cases compared to the reference TGT haplotype, which is consistent with the direction of the individual protective associations found for rs2236757-A and rs9984273-C; however, the linkage disequilibrium analysis indicated only weak LD between rs1051393 and rs2236757 and no significant LD with either rs1051393 or rs9984273. The GAC haplotype, absent from ICU patients, gave a markedly low continuity-corrected estimate (OR = 0.03), but this rests on a zero cell and on EM-inferred frequencies and is likewise hypothesis-generating.

Therefore, the resulting haplotypes mostly represent independent assortment of the underlying alleles rather than a co-inherited functional unit (see Section 3.3). As discussed previously, the estimated effect of the TAC haplotype was based on a small sample size of carriers, had a wide confidence interval, and did not pass multiple testing corrections across the eight haplotypes examined; thus, the TAC estimate should be regarded as an exploratory observation that describes rather than establishes evidence of a biologically coherent, additive, or interactive effect of the three loci.

Several limitations exist with regard to how these results can be interpreted. As the hospitalized/ICU subgroup (*n* = 46) is relatively small, there exists an inherent limitation to the statistical power of the analysis and an increased likelihood for both false-negative and false-positive findings, especially concerning the rs1051393 variant and for the haplotype-based associations (most notably TAC and GAC, which have extremely low carrier rates and therefore represent mere exploratory trends rather than definitive findings); therefore, the identified associations should be viewed as preliminary.

Additionally, TAC association did not remain significant after adjustment for multiple testing over the haplotype comparisons, so greater caution is required when interpreting it. The recruitment of study participants occurred from a single private biological analysis laboratory. This circumstance may lead to some degree of selection/referral bias, which could potentially reduce the generalizability of the cohort’s demographics compared to the overall population in Tunisia; such biases may also partially account for the unique demographic characteristics identified above (younger age and reduced male dominance among those hospitalized). Fourthly, since all samples were collected prior to the widespread implementation of COVID-19 vaccination throughout Tunisia in 2021, these findings may not apply to current circulating SARS-CoV-2 variants or to vaccinated individuals. Fifthly, population stratification was not formally evaluated; therefore, it was impossible to use either ancestry-informative markers or principal component analysis of genome-wide genotype data to identify or adjust for substructure within the Tunisian cohort. Furthermore, a certain amount of stratification cannot be completely ruled out. Sixthly, this study did not systematically collect or screen for secondary bacterial/viral/fungal co-infections.

Secondary infections are a well-recognized cause of critical illness in hospitalized COVID-19 patients; because type I interferon signaling mediated by *IFNAR2* influences antibacterial/antifungal immunity, it is not possible to rule out the possibility that a portion of the observed differences in severity is attributable to variations in susceptibility to secondary infections as opposed to or in addition to an influence upon SARS-CoV-2-associated severity. Seventhly, this study represents a candidate-gene association study without any direct functional validation (i.e., *IFNAR2* expression/protein levels within our cohort).

Consequently, the mechanistic explanations put forth herein are purely speculative until they are validated experimentally. Confirmation of these results will require replication in larger, independent, multicenter, and genetically diverse cohorts ideally combined with functional studies and systematic documentation of co-infection status.

## 5. Conclusions

Our candidate-gene association study shows preliminary evidence that genetic variations at *IFNAR2* may contribute to the risk of developing critical COVID-19 in Tunisians. The SNPs rs2236757 and rs9984273 were found to be associated with reduced risks of developing critical disease. Conversely, the SNP rs1051393 was not independently associated with critical disease. We also observed an association for the corresponding haplotypes; specifically, the TAC haplotype appears to represent a potentially protective haplotype when compared to the reference haplotype TGT, and the GAC haplotype, absent from ICU patients, showed a low continuity-corrected estimate that is based on a zero cell. However, because of the small sample size (*n* = 46), the case-control study design, the single-center recruitment process, the retrospective nature of the study, and the absence of functional validation and of systematic co-infection screening, these findings can only serve as hypothesis-generating.

Furthermore, since the samples were obtained from non-vaccinated patients who were recruited during the year 2021, the generalizability of these findings to the current epidemiology and to currently circulating viral variants may be limited. To confirm and validate the associations observed in this study, additional large-scale independent and multicenter cohort studies will need to be conducted.

Additionally, functional studies investigating *IFNAR2* expression and signaling in response to COVID-19 infection are needed to identify the underlying mechanisms prior to considering any potential clinical or therapeutic applications.

## Figures and Tables

**Table 1:** Demographic and clinical characteristics of the study participants stratified by disease severity.

Characteristics	Non-Hospitalized (*n* = 367)	Hospitalized (ICU) (*n* = 46)	*p*-Value
Age (mean ± SD)	51.5 ± 16.65	48.5 ± 17.2	0.251 *
Sex Male Female			0.871 **
	125 (34.1%)	15 (32.6%)	
	242 (65.9%)	31 (67.4%)	
Tobacco	81 (22.1%)	10 (21.7%)	1 **
Hookah	40 (10.9%)	2 (4.3%)	0.203 **
Alcohol	45 (12.3%)	3 (6.5%)	0.333 **
Comorbidities			
Cardiovascular diseases	57 (15.5%)	5 (10.9%)	0.514 **
Diabetes	58 (15.8%)	3 (6.5%)	0.122 **
Cardiovascular diseases + Diabetes	42 (11.4%)	6 (13%)	0.806 **
Asthma	4 (1.1%)	2 (4.3%)	0.136 **
Thyroid	20 (5.4%)	2 (4.3%)	1 **
Other diseases	22 (6%)	4 (8.7%)	0.514 **
No diseases	164 (44.7%)	24 (52.2%)	0.350 **

Data are presented as mean ± SD or *n* (%). ICU: intensive care unit. * Student’s *t*-test. ** Chi-square test or Fisher’s exact test, as appropriate.

**Table 2:** Genetic association of the three variants within *IFNAR2* gene under the different genetics models (codominant, dominant and recessive).

Variant	Model	Genotype	Non-Hospitalized (*n* = 367)	Hospitalized ICU (*n* = 46)	OR (95% CI) ^a^	*p-Value* ^b^	OR (95% CI); Adjusted *p-Value* ^c^	AIC	BIC	FDR ^d^	*p-Value* Inter ^e^
rs1051393 Alleles	Codominant Dominant Recessive	TT TG GG TT GG + TG TT + TG GG G T	164 (44.7%) 151 (41.1%) 52 (14.2%) 164 (44.7%) 203 (55.3%) 315 (85.8%) 52 (14.2%) 255 (35%) 479 (65%)	22 (47.8%) 21 (45.6%) 3 (6.5%) 22 (47.8%) 24(52.2%) 43 (93.5%) 3 (6.5%) 27 (29%) 65 (71%)	1.00 1.04 (0.55–1.96) 0.43 (0.12–1.50) 1.00 0.88 (0.48–1.63) 1.00 0.42 (0.13–1.41)	0.29 0.69 0.12	1.00 1.05 (0.55–1.99); 0.24 0.41 (0.12–1.42) 1.00; 0.67 0.87 (0.47–1.62) 1.00; 0.094 0.40 (0.12–1.33)	294.5 295.1 292.5	314.6 311.2 308.6	0.27 0.67 0.12	0.3
rs2236757 Alleles	Codominant Dominant Recessive	AA AG GG GG AA + AG GG + AG AA G A	65 (17.7%) 164 (44.7%) 138 (37.6%) 138 (37.6%) 229 (62.4%) 302 (82.3%) 65 (17.7%) 440 (60%) 294 (40%)	3 (6.5%) 14 (30.4%) 29 (63%) 29(63%) 17 (37%) 43 (93.5%) 3 (6.5%) 72 (78%) 20 (22%)	0.22 (0.06–0.75) 0.41 (0.21–0.80) 1.00 1.00 0.35 (0.19–0.67) 1.00 0.32 (0.10–1.08)	0.0028 0.001 0.034	**0.16 (0.05–0.58)** **0.38 (0.19–0.76); 7 × 10^−4^** 1.00 1.00 **0.31 (0.16–0.60); 4 × 10^−4^** 1.00 **0.25 (0.07–0.86); 0.01**	282.7 282.6 * 288.7	302.8 298.7 * 304.8	0.00315 0.0036 0.018	0.36
rs9984273 Alleles	Codominant Dominant Recessive	TT TC CC TT CC + TC TT + TC CC T C	103 (28.1%) 174 (47.4%) 90 (24.5%) 103 (28.1%) 264 (71.9%) 277 (75.5%) 90 (24.5%) 380 (51.8%) 354 (48.2%)	23 (50%) 18 (39.1%) 5 (10.9%) 23 (50%) 23 (50%) 41 (89.1%) 5 (10.9%) 64 (70%) 28 (30%)	1.00 0.46 (0.24–0.90) 0.25 (0.09–0.68) 1.00 0.39 (0.21–0.73) 1.00 0.38 (0.14–0.98)	0.0061 0.0033 0.026	1.00 **0.49 (0.25–0.97); 0.009** **0.25 (0.09–0.70)** 1.00 **0.41 (0.22–0.76); 0.0055** 1.00 **0.37 (0.14–0.96); 0.023**	287.8 287.6 * 290.1	308 303.7 * 306.2	0.018 0.0165 0.0345	0.36

* Low Akaike information criteria (AIC) and Bayesian information criteria (BIC). ^a^ The odds ratios (OR (95% CI) from Fisher’s exact test. ^b^ *p*-values from Fisher’s exact test. ^c^ The odds ratios and the *p*-values were from logistic regression models adjusted for age and sex. ^d^ Benjamini–Hochbergmethod. ^e^ *p*-value from the interaction test of the sex with the variant. *p*-values below the significance threshold (0.05) of the best model are highlighted in bold.

**Table 3:** Haplotype frequencies and association with critical form of COVID-19.

Rank	rs1051393	rs2236757	rs9984273	Total Frequency	Non-Hospitalized (*n* = 367)	Hospitalized (ICU) (*n* = 46)	OR (95% CI)	*p*-Value	Global Haplotype Association *p*-Value
1	T	G	T	25.99%	24.20%	39.03%	1.00 (reference)	-	0.00018
2	T	G	C	24.67%	24.84%	25%	0.62 (0.33–1.17)	0.14	
3	G	A	T	13.43%	13.47%	15.11%	0.53 (0.26–1.11)	0.095	
4	G	A	C	9.38%	10.36%	0%	0.03 (0.002–0.53) *	0.00001	
5	T	A	T	8.42%	8.83%	5.37%	0.52 (0.18–1.49)	0.22	
6	T	A	C	6.79%	7.40%	1.25%	0.11 (0.02–0.86)	**0.036**	
7	G	G	T	5.92%	5.28%	10.05%	1.13 (0.46–2.81)	0.79	
8	G	G	C	5.41%	5.63%	4.18%	0.74 (0.20–2.71)	0.65	

ICU, intensive care unit; OR, odds ratio; CI, confidence interval, *p*-value below the significance threshold (<0.05) is highlighted in bold. * Odds ratio and 95% CI for the rare GAC haplotype (0% in ICU cases) were calculated using Haldane–Anscombe continuity correction (adding 0.5 to zero-count cells) to yield a finite risk estimate.

## Data Availability

The data can be made available by contacting the corresponding author.
